# Microbe Profile: Cellvibrio japonicus: living the sweet life via biomass break-down

**DOI:** 10.1099/mic.0.001450

**Published:** 2024-04-03

**Authors:** Jeffrey G. Gardner

**Affiliations:** 1 Department of Biological Sciences, University of Maryland – Baltimore County Baltimore, Maryland, USA

**Keywords:** biotechnology, carbohydrate active enzyme, *Cellvibrio japonicus*, polysaccharide degradation, saprophyte

## Abstract

*Cellvibrio japonicus* is a saprophytic bacterium proficient at environmental polysaccharide degradation for carbon and energy acquisition. Genetic, enzymatic, and structural characterization of *C. japonicus* carbohydrate active enzymes, specifically those that degrade plant and animal-derived polysaccharides, demonstrated that this bacterium is a carbohydrate-bioconversion specialist. Structural analyses of these enzymes identified highly specialized carbohydrate binding modules that facilitate activity. Steady progress has been made in developing genetic tools for *C. japonicus* to better understand the function and regulation of the polysaccharide-degrading enzymes it possesses, as well as to develop it as a biotechnology platform to produce renewable fuels and chemicals.

## Graphical abstract

Transmission Election Microscopy (TEM) image of *Cellvibrio japonicus* from a Hitachi HT7800 set to 100kV and stained with NanoW. The scalebar depicts 1µm. The polysaccharide diagrams shown are examples of substrates that the bacterium is adept at degrading. *C. japonicus* possesses an impressive array of carbohydrate active enzymes to cleave the glycosidic bonds of many plant, animal, and fungal polysaccharides.

## Taxonomy

Domain: *Bacteria*, Phylum: *Pseudomonadota*, Class: *Gammaproteobacteria*, Order: *Cellvibrionales*, Family: Callvibrionaceae, Genus: *Cellvibrio*, Species: *Cellvibrio japonicus*. The type strain is *C. japonicus* Ueda107.

## Properties

*Cellvibrio japonicus* was isolated in 1948 from field soil in Saitama Prefecture, Japan. It is a Gram-negative, non-spore forming, rod-shaped bacterium, and motile via one polar flagellum. While very poor anaerobic growth has been reported, all published physiological studies of *C. japonicus* characterize it as an aerobe with an optimum growth temperature of 30 °C, pH optimum of 7.5, and salinity tolerance up to 3 % (w:v) NaCl. *C. japonicus* excretes a green fluorescent compound that is visualizable under UV light on a minimal media agar plate. The hallmark feature of *C. japonicus* is that it can degrade diverse environmental polysaccharides of plant and animal origin [1].

## Genome

*C. japonicus* has a 4.5 Mb genome with a G+C content of 52 % that is predicted to encode 3790 proteins, with ~66 % having been assigned a cellular function [2]. There is one Tn3 and one Tn7 transposon found in the genome, along with a single 4.7 kb CRISPR array. The sequences of two spacers in the array match *Pseudomonas* phage 73 (PA73), while the remaining spacers did not match any existing sequences in NCBI databases. The *C. japonicus* genome encodes for 130 predicted glycoside hydrolases (GH), 46 glycoside transferases (GT), 14 polysaccharide lyases (PL), 16 carbohydrate eseterases (CE), two lytic polysaccharide monooxygenases (LPMO), and 17 carbohydrate-binding module-containing proteins that do not have an assigned function. These predicted carbohydrate active enzymes are encoded by ~6 % of the genome, however few of these genes are clustered together, which is markedly different compared to other Gram-negative polysaccharide degraders (e.g*. Bacteriodes thetaiotaomicron*) where polysaccharide utilization loci (PULs) are the norm. Three other strains of *C. japonicus* have been sequenced, which were derived from experiments where the bacterium was grown using rare α-diglucosides (kojibiose, nigerose, and isomaltose). Across these three strains 36 to 60 gene sequences were different from the Ueda107 type strain, with most of the changes being gene truncations.

## Phylogeny

*C. japonicus* is currently one of 11 *Cellvibrio* species, however it has undergone two major nomenclature changes since its isolation and initial characterization. When first published in 1952, the bacterium was classified as *Pseudomonas fluorescens* subsp. *cellulosa*, however a reassessment of the bacterium in 1995 suggested a name change to *Pseudomonas cellulosa* based largely on substrate utilization and phospholipid fatty acid analysis. This name was used for a few years until finally in 2003 molecular genetic analysis reclassified the bacterium as belonging to the *Cellvibrio* genus and assigned the name *C. japonicus*, with the species name being derived from the country where it was first isolated. In the 2008 paper that reported the complete genome sequence, it was noted that *C. japonicus* is closely related to *Sacarophagus*, *Microbulbifer*, and *Teridinibacter* spp., all of which contain members that are proficient at complex polysaccharide degradation [2].

## Key features & discoveries

The most notable feature of *C. japonicus* is the robust ability to degrade complex and recalcitrant polysaccharides, such as those found in lignocellulose, crustacean and insect shells, and fungal cell walls. The published data strongly suggested that *C. japoncius* is a polysaccharide utilization specialist, not only due to its robust growth using substrates like insoluble cellulose and chitin, but also very poor growth in rich media using peptides or amino acids as sole carbon sources [3,4].

The bacterium’s carbohydrate active enzymes (CAZymes) have been studied since the 1950s with considerable work on the cellulase, xylanase, mannanase, and arabainase degradative systems. In addition to catalytic domains, CAZymes from *C. japonicus* often also have carbohydrate binding modules, and considerable work has deciphered structure/function connections and how these protein domains specifically bind polysaccharide substates [5]. Lytic polysaccharide monooxygenases were an exciting discovery in the field of polysaccharide degradation, and characterization of the two LPMOs from *C. japonicus* helped to establish important enzymatic and physiological properties of this enzyme class [6].

Within the past ten years a genetic system for *C. japonicus* has been developed and employed to characterize the physiological roles of the large number of CAZymes the bacterium possesses. One early discovery was that a Type II Secretion System was essential for the export of *C. japoncus* CAZymes [7]. More recent work using RNAseq and other systems biology approaches has uncovered that *C. japonicus* primarily regulates the expression for CAZyme-encoding genes via substrate detection rather than by growth rate. Additionally, *C. japonicus* has been noted to possess a vast array of TonB-dependent transporters, and studies in other bacteria proficient in polysaccharide degradation have suggested that a diverse array of nutrient transporters is one mechanism to remain competitive in environments rich in polysaccharides.

Synthetic biology studies have demonstrated that *C. japonicus* cellulases expressed in *E. coli* are functional and confer a weak ability to degrade cello-oligosaccharides [8]. Additionally, *C. japonicus* strains have been engineered to produce ethanol from cellulose or rhamnolipids from xylan directly via heterologous expression from a shuttle plasmid [9]. The interest in *C. japonicus* as a synthetic biology platform also resulted in a study where transcriptomic and growth data were used to generate a series of metabolic models to optimize the bacterium for cellodextrin utilization [10]. These reports have shown at the proof-of-concept level that *C. japonicus*, and its enzymes, have the potential for future biotechnology applications in the renewable chemical or biomedical sectors.

## Open questions

Where does *C. japonicus* fit into a complex microbial community, specifically is it an active helper or a peripheral scavenger?

What is the energetic cost (total ATP) of glycoside hydrolase synthesis and export in *C. japonicus*, and how is it offset by sugar recovery?

Can the regulatory circuits that drive carbohydrate active enzyme expression in *C. japonicus* be identified and subsequently altered for the optimum bioconversion of specific polysaccharide-containing substrates?

Why does *C. japonicus* possess >40 TonB-dependent transporters, and what are their specific physiological functions?

How does *C. japoncus* fulfil its nitrogen needs, given that lignocellulose is a nitrogen poor substrate and that the bacterium does not utilize peptides (or amino acids) as sole nitrogen sources?

## Short biography

Jeffrey G. Gardner has researched *Cellvibrio japonicus* physiology and metabolism for over 15 years. His laboratory developed genetic and systems biology tools for the bacterium, as well as methods to measure growth and enzyme activity while *C. japonicus* is actively degrading complex insoluble polysaccharide substrates.

## Abbreviation

CAZyme: carbohydrate active enzyme
